# Development of a Sex-Specific Marker for the Chinese Hooksnout Carp *Opsariichthys bidens* Günther, 1873 Based on Whole-Genome Resequencing and Bulked Segregant Analysis

**DOI:** 10.3390/ani15213164

**Published:** 2025-10-31

**Authors:** Feng Lin, Ruobing Zhao, Maosheng Miao, Yuchen Wang, Ning Lei, Dewen Ding, Rongrong Wang, Shan Ouyang, Xiaoping Wu, Chunhua Zhou

**Affiliations:** 1 Key Laboratory of Healthy Freshwater Aquaculture, Ministry of Agriculture and Rural Affairs, Zhejiang Institute of Freshwater Fisheries, Huzhou 313001, China; wwlinfeng@163.com (F.L.); faywallace@163.com (Y.W.); 2 School of Life Sciences, Nanchang University, Nanchang 330031, China; zhao741211@163.com (R.Z.); 18393847876@163.com (R.W.); liminghuadi@163.com (S.O.); xpwu@ncu.edu.cn (X.W.); 3 Shangrao Agriculture, Forestry and Water Science Research Center, Shangrao 334000, China; zmmsheng@163.com (M.M.); yleining@163.com (N.L.); ncuskdingdewen@163.com (D.D.)

**Keywords:** *Opsariichthys bidens*, bulked segregant analysis, genome resequencing, sex-specific marker

## Abstract

Sex-specific markers are important basic tools for the sex-controlled breeding of farmed fish. Here, we aimed to develop a rapid yet accurate, cost-effective method for determining the genetic sex of the Chinese hooksnout carp (*Opsariichthys bidens*), a freshwater fish. Using whole-genome resequencing technology, along with bulked segregant analysis (BSA) and chromosome quotient (CQ) methods, sex-specific regions were screened, and corresponding primers were designed to validate the screening results. A total of 45 sex-specific regions were successfully screened through BSA sequencing and CQ analysis, and 50 pairs of primers were designed for use in the screening verification. The Mar28 primer pair showed stable sex specificity in multiple populations of *O. bidens*, accurately distinguishing male from female individuals. This primer pair amplified two bands (509 and 814 bp) in males, but only one band (509 bp) in females. The genetic sex identification method established here provides a theoretical basis for studying the mechanism of sex determination in *O. bidens*, has implications for the monosex culture and molecular breeding of *O. bidens*, and has significant scientific and practical value.

## Simple Summary

This study developed and validated a rapid, accurate, and cost-effective genetic sex identification method for the freshwater fish *Opsariichthys bidens*. Using whole-genome resequencing and the chromosome quotient approach, 45 male-specific genomic regions were identified. Among 50 designed primer pairs, the Mar28 primer pair consistently distinguished males from females across multiple populations, amplifying two bands in males and only one band in females. This marker provides a practical tool for monosex breeding and aids further research on sex determination in *O. bidens*.

## 1. Introduction

Fish species have complex modes of sex determination, namely genetic sex determination, environmental sex determination, or a combination of both [1,2]. The fish sex chromosome system is intricate and diverse, harboring almost all the vertebrate sex chromosome types, the two most common being male heterozygosity (XX/XY) and female heterozygosity (ZW/ZZ) [3]. Many fish species are sexually dimorphic, with males and females differing markedly in growth rate. For example, males of *Mastacembelus armatus* [4] and *Channa argus* [5] grow faster than females. Conversely, females of *Siniperca chuatsi* [6] and *Siniperca scherzeri* [7] grow faster than conspecific males. Hence, understanding how sex is determined in fish is critically important in aquaculture because it enables the production of all-male (or all-female) juveniles (monosex culture), which increases the yield per unit of culture. Sex control and single-sex culture have long been key topics in fisheries and aquaculture worldwide, and sex-specific molecular markers have always been at the forefront of fish breeding research. The international community has long sought sex-specific molecular markers and reliable genetic sex determination methods for different fish species [8].

Whole-genome resequencing involves the resequencing of the entire genome of a species at the chromosome level. Comparison with the reference genome permits inherent variation in the whole genome to be detected and yields molecular genetic information for individuals or populations [9]. Compared with de novo genome assembly, whole-genome resequencing has various advantages, including low sequencing costs and the simplicity of bioinformatics analyses; it has thus become the main approach for the development of sex identification markers [9,10]. This method has yielded sex-specific markers for various aquaculture fish species, such as *Megalobrama amblycephala*, *Platichthys stellatus*, and *Mastacembelus armatus* [9,10,11,12,13]. Bulked segregant analysis (BSA) is based on genome-wide resequencing technology and can quickly identify specific genes as well as regions of variation within the genome, in addition to screening out markers associated with certain phenotypes; hence, it is a simple and efficient trait mapping technique [14]. Sex identification markers have been developed successfully for fish such as *Micropterus salmoides* and *Trachinotus ovatus* using BSA [15,16].

The Chinese hooksnout carp (*Opsariichthys bidens*) in the order Cypriniformes, family Cyprinidae, is named because of the gap and protrusions in its upper and lower jaw, which resemble a horse’s mouth. This freshwater fish is widely distributed in East Asia and is often found in clear, fast-flowing shoals, sandy bottom streams, and river tributaries, where it feeds on small fish, shrimp, and aquatic insects [17]. Owing to its rapid growth and plump, tender flesh, *O. bidens* is an economically important aquaculture species with high aquaculture value. This species shows sexual dimorphism, with males growing faster than females, and males have a blue–green pattern during the breeding period with high ornamental value; females have an ordinary body color [18,19]. Therefore, the male-only culture of *O. bidens* could significantly improve both its yield and economic benefits. However, because males and females are not readily distinguishable prior to adulthood, rapid and efficient sex identification markers are needed. Earlier work reported that RAPD primers derived from gonadal DNA could amplify bands distinguishing the sex of *O. bidens*, but this requires gonadal tissue, which is difficult to extract from young fish [20]. The complete genome of *O. bidens* has been sequenced [18,21], and its XX/XY sex determination system [22] is conducive to the development of new sex determination techniques. Recently, genome-wide resequencing technology was used in a comparative study of the male and female genomes of *O. bidens*. A male-specific insertion fragment was detected, from which a PCR-based sex identification method was then constructed; the marker was amplified in males only, not in females [23]. A sex-linked SNP marker in *O. bidens* has been reported, which is heterozygous in males and homozygous in females, and is located within an exonic region [24]. Nevertheless, more sex identification markers are needed to aid sex-controlled breeding and facilitate further research on the mechanisms of sex determination and differentiation in *O. bidens*.

In the present study, mixed pool sequencing was performed on male and female *O. bidens* based on whole-genome resequencing. The chromosome quotient (CQ) method was used to screen for regions with sex-specific sequences. The CQ method maps resequencing reads to a reference genome and then slides a window across the alignment to count the number of mapped reads in each bin. Multiple pairs of primers were designed for each region, and their sex specificity in the *O. bidens* population was verified through PCR amplification. This yielded sex identification markers for *O. bidens*, which amplified two bands in males but just one band in females. Our findings provide a theoretical reference for future research on sex determination mechanisms and the sex-controlled breeding of *O. bidens* and have practical implications for enhancing breeding management.

## 2. Materials and Methods

### 2.1. Sample and DNA Extraction

The samples used for resequencing and primer screening were derived from an artificially bred population with a known genetic background in Jiangxi Province. In June 2023 (breeding season: June–September), 25 males (body length 13.02 ± 0.82 cm, body weight 24.94 ± 4.69 g) and 25 females (body length 12.24 ± 0.54 cm, body weight 19.39 ± 1.69 g) were selected for sequencing; males and females were distinguished via morphology and inspection of their gonads after dissection. The tail fin of each fish was immediately flash-frozen and then stored at ultralow temperatures until the DNA was extracted. This DNA was subsequently used to create a mixed pool for BSA sequencing. The fish populations used for primer validation were collected from Jiangxi, Zhejiang, and Fujian during the breeding season (20 male and 20 female adults per population). To extract the genomic DNA of *O. bidens*, the BayBiopure magnetic bead animal tissue genomic DNA extraction kit was used (BayBio, Guangzhou, China). The amount of genomic DNA was determined using a NanoDrop 2000 spectrophotometer (Thermo Fisher Scientific, Waltham, MA, USA), and its quality was assessed via 0.7% agarose gel electrophoresis. Care of animals was in compliance with the guidelines of the Animal Experiment Committee, Zhejiang Institute of Freshwater Fishery (ZJIFF20230603).

### 2.2. Library Construction and BSA Sequencing

The qualified genomic DNA samples of female and male *O. bidens* were mixed in equal amounts, resulting in two genomic DNA pools for females and males, respectively. Their DNA samples were broken into short fragments (200–400 bp each), using a Covaris ultrasonic breaker (50 hertz, 20 min) (Covaris, Woburn, MA, USA). After undergoing terminal repair, poly(A) tails and sequencing adapters were attached, and this was followed by purification and PCR amplification; the resulting PCR products were then cyclized to complete the library construction. Next, the DNA concentration was determined using a Qubit 3.0 fluorometer (Life Technologies, Carlsbad, CA, USA), and the library was quantified. The library’s insert size was detected using an Agilent 2100 Bioanalyzer (Agilent, Santa Clara, CA, USA) to confirm that the size of the inserted fragment was as expected. The precise effective concentration of the library was subsequently determined using qPCR to ensure the overall quality of the library. Finally, each pooled library was sequenced on the DNBSEQ platform, with each pool producing approximately 20 GB of data.

### 2.3. Initial Data Processing and Quality Assessment

Initial image data files were converted into raw sequencing reads via base calling, and clean reads were generated using fastp (v0.20.1) for quality control with six filtering criteria [25]. The obtained clean reads from all samples were aligned to the *O. bidens* reference genome using BWA software (v0.7.17) [26]. The alignment results were converted from SAM files into sorted BAM files, from which any duplicates were removed using SAMtools software (v1.19) [27]. The alignment rate and coverage were then calculated using a Python (v3.12) script.

### 2.4. Screening of Sex-Specific Regions

Based on the resulting data (BAM file) generated by BSA and the reference genome (GWHBJYU00000000, https://ngdc.cncb.ac.cn/gwh/Assembly/26062/show, accessed on 30 September 2023), the chromosome quotient (CQ) was used to screen the male-specific sequence regions. The genome of male *O. bidens* was screened (window size = 500 bp, step = 1000 bp), and the CQ value of each sliding window was calculated as follows: CQ = Hf/Hm (where Hm is the number of reads in the reference genome of the male mixed-pool comparison; Hf is the number of reads in the reference genome of the female mixed-pool comparison). The male-specific regions with a CQ < 0.2 and an Hm > 30 were retained. These thresholds were based on the references from published literature [28]. Overlapping windows identified during screening were merged. Integrative Genomic Viewer (v2.16.2) was used to compare reads in the selected regions and further confirm male-specific reads.

### 2.5. Design of Primers for Sex-Specific Regions and Their Screening Validation

Flanking sequences (1 kb upstream and downstream) of each sex-specific region were extracted, and common PCR amplification primers were designed using Primer Premier software (v6.0). These primers were screened via two rounds of PCR amplification. First, the genomic DNA of three males and three females of *O. bidens* served as the template for the preliminary screening of primers. For those primers with either unclear amplification bands or no target bands, the annealing gradient was adjusted to find the optimal annealing temperature until clear and complete target bands were obtained. For primers that met the expected specificity for the target region, sex specificity was further validated using the *O. bidens* populations from Jiangxi, Zhejiang, and Fujian (20 males and 20 females per population). Every PCR amplification was performed in a 25 μL reaction system, consisting of 12.5 μL of 2 × Rapid Taq Master Mix, 1.0 μL of each F/R primer (10 μmol/L), 1.5 μL of DNA template, and 9.0 μL of ddH_2_O. A negative control using ddH_2_O as the template was also included. The PCR amplification program was as follows: initial denaturation at 94 °C for 5 min; 30 cycles of denaturation at 94 °C for 30 s, annealing at 53–59 °C for 30 s, followed by an extension at 72 °C for 2 min; and a final extension at 72 °C for 10 min. Samples were then stored at 4 °C. The primers’ specificity was confirmed by 1% agarose gel electrophoresis of the PCR products. The PCR products of primers that targeted the expected sex-specific region and yielded sex-differentiating bands were purified and ligated into the PMD-18 vector for cloning. The ligation mixtures were transformed into DH5α *Escherichia coli* competent cells, putative positive colonies were selected, and these were sequenced by Shengong Bioengineering (Shanghai, China) Co., Ltd. Male and female sequences of *O. bidens* were analyzed in SnapGene software (v6.0).

## 3. Results

### 3.1. Sample Information

**Figure 1:**
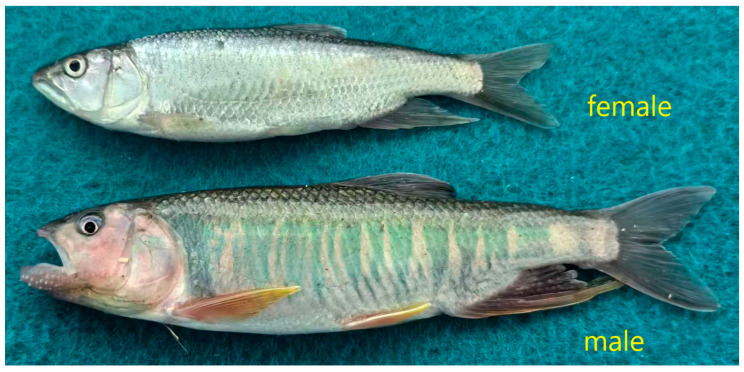
Male and female *Opsariichthys bidens*.

The genetic background, specifications, and age information of the male and female fish populations used for resequencing and primer validation are shown in Table 1. Photographs clearly illustrating the differences in sexual dimorphism between the sexes are shown in Figure 1.

The genomic DNA of 25 male and 25 female fish was detected using a NanoDrop spectrophotometer. Their average DNA concentration was 275.92 ng/μL, with an OD260/OD280 between 1.8 and 2.0. The results of 1% agarose gel electrophoresis showed clear DNA bands and no serious degradation. This indicated that the collected genomic DNA of *O. bidens* was of high quality and suitable for BSA-seq library preparation.

### 3.2. Sequencing Data Statistics

From the BSA sequencing, raw data totaling 21.54 GB and 28.20 GB were generated for the mixed pools of male and female *O. bidens*, respectively. After undergoing data filtering and quality control, effective data amounting to 20.73 GB and 26.98 GB were obtained for males and females, respectively. Their Q20 was 98.96% and 99.03%, their Q30 was 95.64% and 95.92%, and their GC content was 38.95% and 38.94%, respectively (Table 2). After duplicate reads were removed, 142,668,885 and 186,590,651 sequences were obtained from the male and female mixed pools, respectively. Of those, 141,763,631 and 185,468,646 sequences were aligned to the *O. bidens* reference genome, yielding alignment rates of 99.37% and 99.40%, genome coverage of 96.33% and 96.44%, and an average sequencing depth of 24.22× and 31.46×.

### 3.3. Screening and Validation of Sex-Specific Markers

**Figure 2:**
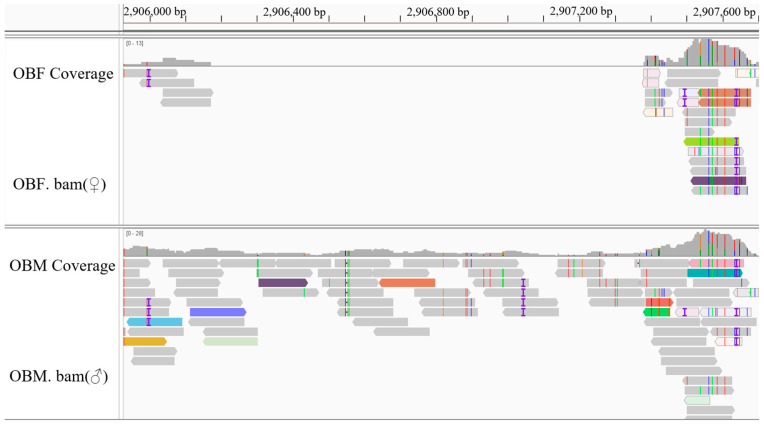
Visualization of the sex-specific region for the primer Mar38. The target region was located on chromosome 20 (genomic coordinates: chr20: 2,906,224–2,907,256), spanning 1032 bp. The analysis was extended to include 300 bp of the flanking sequence on both sides of this core interval.

**Figure 3:**
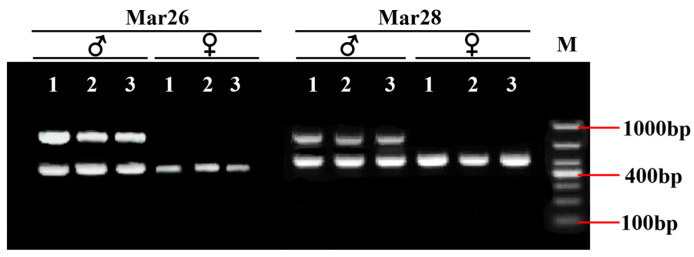

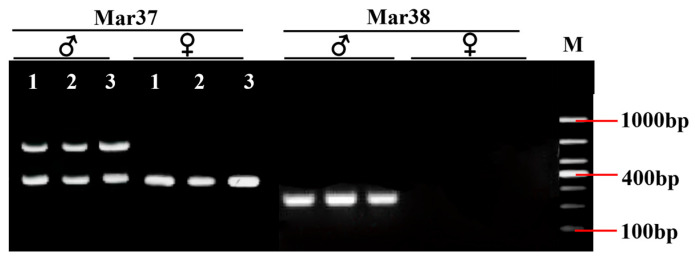
PCR amplification electrophoretogram of four pairs of primers in six *Opsariichthys bidens* individuals.

**Figure 4:**
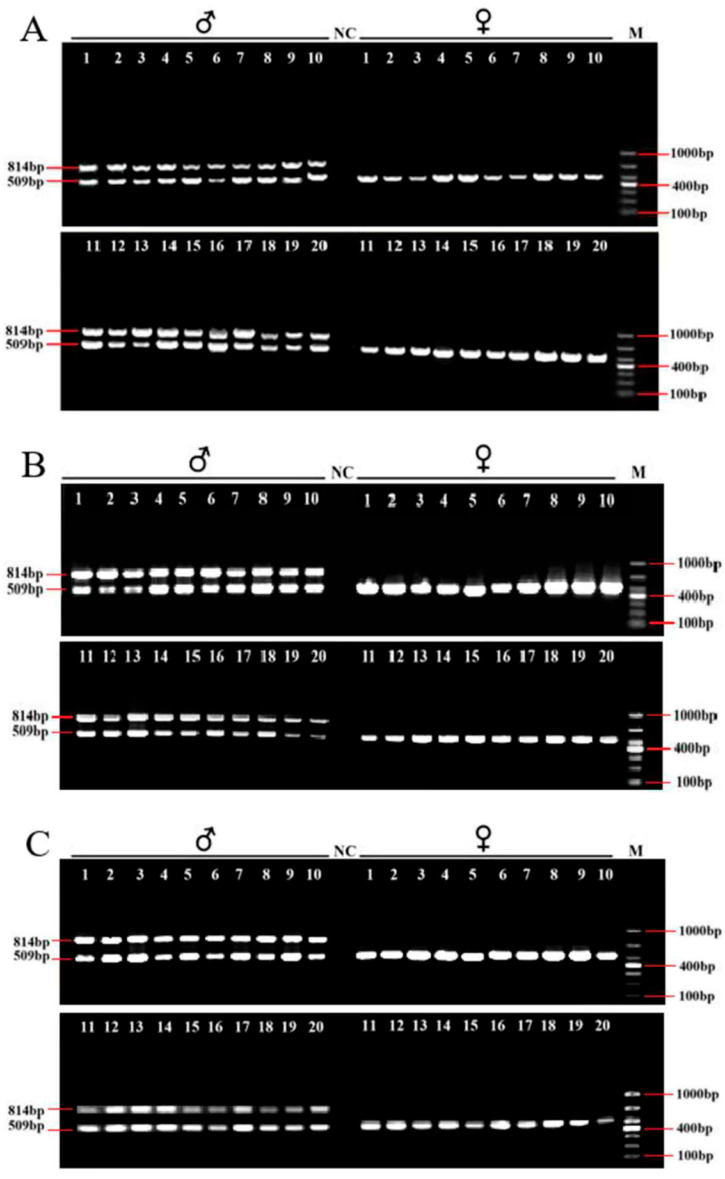
Amplified PCR electrophoretogram of Mar28 in 40 *Opsariichthys bidens* individuals from Jiangxi (**A**), Zhejiang (**B**), and Fujian (**C**). NC, the negative control. M, the DL1000 Marker.

**Figure 5:**
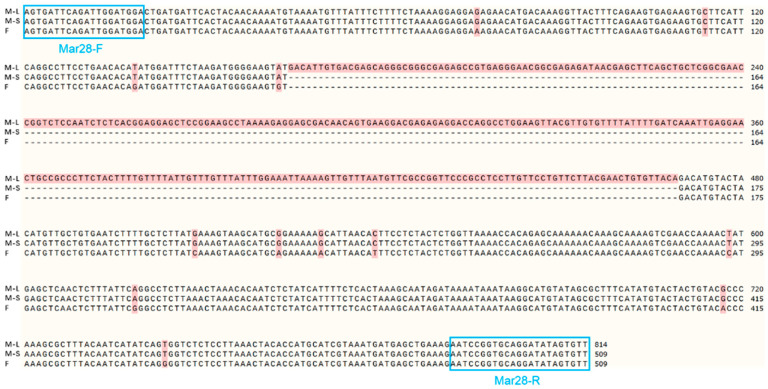
Sequence alignment of the Mar28 amplified fragments in male and female *Opsariichthys bidens*. M-L denotes the long sequence in males, while M-S denotes the short sequence in males; F refers to the female sequence, with “—” indicating a missing sequence. Highlighted in red are the base differences between the male and female sequences; the blue box shows the position of the primer pair.

After the IGV examination, 45 sex-specific regions (CQ < 0.2) were found (Figure 2). For these, 50 pairs of primers were designed according to their specific regional flanking sequences (Table 3). Three males and three females were randomly selected to screen those 50 primer pairs in the first PCR amplification round: the amplification bands of four pairs (the primers Mar26, Mar28, Mar37, and Mar38) showed pronounced differences between males and females (Figure 3). Hence, these four pairs of primers were subjected to further screening in a second PCR amplification round; that is, 40 samples (20 male and 20 female adult fish per population) from each of the Jiangxi, Zhejiang, and Fujian populations were used for verification. These results revealed that Mar28, located on chromosome 8, amplified two bands in males but only a single band in females in all three *O. bidens* populations (Figure 4 and Table 3). In contrast, the amplified bands of the other three primers failed to yield a difference between the sexes in all individuals. Further sequencing of the Mar28 primer amplification products showed that Mar28 amplified two respective bands of 509 and 814 bp in males, while a single band of 509 bp was amplified in females (Figure 5).

## 4. Discussion

Fish are the mainstay of aquaculture, and fish farming is an important industry for ensuring China’s national food security. China is home to a wide variety of farmed fish species, and the development of sex-specific molecular markers can greatly aid the aquaculture industry. Research on sex control and monosex breeding techniques is critically important, given that many cultured fish exhibit sexual differences. *O. bidens* is an omnivorous species, although it clearly prefers consuming animals and will only consume plants when prey is scarce. Males grow faster than females and display striking coloration during the breeding period. The breeding of all-male fish can thus aid *O. bidens* breeding programs. Determining the genetic sex of *O. bidens* is crucial for achieving a monosex culture. Sex-specific markers are also important for studying the mechanisms of sex determination and differentiation in aquaculture and fisheries.

RAPD, SSRs, AFLPs, and other methods have been successfully used to develop sex identification markers for a variety of fish [29,30,31]. However, these molecular markers have several drawbacks, such as their low efficiency and poor repeatability [32]. In our study, we utilized BSA to address these limitations. BSA allows for the pooling of DNA from individuals with extreme phenotypes, which significantly enhances the signal-to-noise ratio in genetic mapping [33]. This approach enabled us to focus on the most informative genetic markers, thereby increasing the efficiency of identifying trait-associated loci. Furthermore, by using a larger number of individuals per bulk, we improved the repeatability of our results, as the genetic noise from individual variation was minimized. This concrete application of BSA in our study demonstrates its effectiveness in overcoming the challenges posed by traditional methods. Sex identification markers developed via the BSA technique are also being increasingly applied to fish species [34]. A pair of sex markers was selected for *Channa argus* via SSR and BSA, and differential bands could be amplified in most females, but not males [35]. Combining BSA analysis with the resequencing of multiple individuals can eliminate individual differences, provide greater genome coverage of genome-wide information, and reduce the probability of false positives [32]. Using this approach, six male-specific markers were recently developed in *Spinibarbus hollandi* [36]. Some researchers have used the combination of BSA and CQ methods to screen 279 sex-specific regions in *Oryzias curvinotus*, from which two sex markers were developed [28].

The Mar28 marker in our study was obtained by comparing and analyzing genomic resequencing data of female and male *O. bidens* derived from divergent alleles of the same locus, and this marker flanked a Y-specific insertion within a pseudoautosomal region. The Y chromosome has an additional 305-bp fragment, but its function requires further study. Insertion mutations are a common type of mutation characterized by the incorporation of extra nucleotides or DNA segments into existing sequences, which modifies gene sequences and expression patterns [37]. When such insertions occur within intronic regions, they can disrupt or create novel splice sites, which can affect protein structure and functionality [38]. Similar insertional events have been documented in *Oplegnathus punctatus* [39]. Whereas a previous GWAS by Xu et al. [23] mapped all sex-associated loci in *O. bidens* to chromosome 24, the marker developed here was fully embedded within the Y-chromosome insertion and therefore produced a single, male-specific band. In contrast, our newly developed marker resided on chromosome 8 and spanned the insertion junction; consequently, males yielded two bands, whereas females yielded one.

Mining sex-determining or candidate genes via transcriptome sequencing to develop sex-specific molecular markers is also a highly efficient strategy. De novo transcriptome analysis of the testis and ovary of *O. bidens* revealed that *cyp19a* was highly expressed only during the early stage of oocyte development and was not expressed in the testis, while *vasa* was expressed at significantly different levels in the testis and ovary [40]. Transcriptome sequencing analysis with reads mapped to the genome and RT-qPCR verification revealed that *zp3*, *cyp19a*, *hsd17b1*, and *gdf9* were female-biased genes, while *msh4*, *dmrt1*, *rspo2*, and *kif23* were male-biased genes [41]. Transcriptome sequencing of the testis with genome-mapped reads was performed during four developmental stages of male *O. bidens* to determine the genes and pathways related to testis and sperm formation in *O. bidens* [42]. The discovery of these genes will aid the development of sex-specific markers for *O. bidens*. In this study, no association analysis was conducted between the molecular markers obtained and the sex-specific genes. This will be a goal of our future work.

## 5. Conclusions

In this study, sex-specific regions were selected by comparing the resequencing data of mixed pools of females and males to the reference genome of *O. bidens*. Unique primers were designed for these regions, which were then confirmed and validated through PCR, to obtain a robust sex marker. We developed a rapid, accurate, and cost-effective approach for the genetic sex identification of *O. bidens*. Our empirical results will facilitate the production of monosex populations of *O. bidens* and provide a theoretical basis for future investigations of its sex determination and differentiation mechanisms.

## Figures and Tables

**Table 1:** Information on the populations sampled in this study.

Sample	Body Length	Body Weight	Date of Hatching	Age	Sample Size
Male for BSA	13.02 ± 0.82 cm	24.94 ± 4.69 g	September 2022	10th	25
Female for BSA	12.24 ± 0.54 cm	19.39 ± 1.69 g	September 2022	10th	25
Male from Jiangxi	13.73 ± 0.46 cm	27.95 ± 4.49 g	September 2022	12th	20
Female from Jiangxi	12.72 ± 0.56 cm	21.44 ± 3.00 g	September 2022	12th	20
Male from Fujian	12.80 ± 1.06 cm	35.26 ± 13.86 g	August 2023	12th	20
Female from Fujian	11.85 ± 1.27 cm	24.57 ± 11.77 g	August 2023	12th	20
Male from Zhejiang	16.41 ± 0.65 cm	74.08 ± 9.96 g	September 2023	10th	20
Female from Zhejiang	13.71 ± 1.10 cm	41.49 ± 9.30 g	September 2023	10th	20

**Table 2:** BSA sequencing data statistics.

Sample	Raw Bases	Raw Reads	Clean Bases	Clean Reads	Q20 (%)	Q30 (%)	GC (%)	Reads Passed Filters
male	21,540,728,700	143,604,858	20,734,671,780	143,500,274	98.96	95.64	38.95	143,500,274
female	28,202,680,200	188,017,868	26,978,807,394	187,872,836	99.03	95.92	38.94	187,872,836

**Table 3:** Primer sequences designed for the sex-specific regions of *Opsariichthys bidens*. TM, annealing temperature.

Primer Name	Position	Primer Sequence (5′–3′)	Size (bp)	TM (°C)
Mar1	Chr: 136,963,139–36,965,341	F: GTCCTCCTCAGTGAAGTCATTATAC R: GCTCCTCCAGATGTTGTAGAGA	201	57
Mar2	Chr1: 36,964,139–36,964,341	F: TTCGAAAATCTCAGTGCAGC R: TTGACAGTGAAGCTCCTCCA	386	57
Mar3	Chr1: 36,964,139–36,964,341	F: CTCCACCACATTCTGCAGTT R: TTTGTGAATTGGCAGGAACA	492	59
Mar4	Chr1: 36,964,139–36,964,341	F: ACATGCAAGTGTGGCTTTGA R: ACATGCAAGTGTGGCTTTGA	793	57
Mar5	Chr1: 36,964,145–36,964,340	F: CTCACTCTTGTCATGTCCTCCTCA R: GAGAGTTGAAGGAAGACAGAAGGAC	195	57
Mar6	Chr8: 33,584,367–33,586,928	F: CAGGTTGGACTTCAGAACTTACA R: ACGCAGAACGAGCAGGAA	267	58
Mar7	Chr8: 33,584,367–33,586,928	F: AATGCGGAATCTAAGAGCCAAT R: CAACTGCCTCGGAAGAACAA	530	58
Mar8	Chr8: 33,584,929–33,587,296	F: TTCGTGTTGGCTCGGTCTG R: AGTTCATCTGTCATTATGGCTATCC	554	58
Mar9	Chr8: 33,584,929–33,587,296	F: CAGGTTGGACTTCAGAACTTACA R: ACGCAGAACGAGCAGGAA	267	56
Mar10	Chr8: 33,585,297–33,587,717	F: GGATAGCCATAATGACAGATGAACT R: AGCACCAGAACGACCAGAC	499	58
Mar11	Chr8: 33,585,367–33,585,928	F: TTACCAGCAGCTGCACAATC R: GGAATGCCAAAGTTATCGGA	428	57
Mar12	Chr8: 33,585,367–33,585,928	F: AAGGTTTTTGAGCCTGGGTT R: GGAATGCCAAAGTTATCGGA	488	56
Mar13	Chr8: 33,585,407–33,586,707	F: CCGATAACTTTGGCATTCCACTCG R: AAGGTATGCCCTGAGTTTCAGACC	326	56
Mar14	Chr8: 33,585,407–33,586,707	F: TGTAGTGTTCGTGTTGGCTCGG R: AGTGTCTGTGGGAGCAGTTCAT	576	58
Mar15	Chr8: 33,585,929–33,586,296	F: CCACTCGGTAAGTCTGCTCC R: CGACAAATGGGAGGAAACAG	370	58
Mar16	Chr8: 33,585,929–33,586,296	F: GGCATTCCACTCGGTAAGTC R: CGACAAATGGGAGGAAACAG	376	57
Mar17	Chr8: 33,586,297–33,586,717	F: CTGTTTCCTCCCATTTGTCG R: GTGTCTGTGGGAGCAGTTCA	490	58
Mar18	Chr8: 33,586,297–33,586,717	F: CTGTTTCCTCCCATTTGTCG R: TCATTATGGCTATCCCGGAC	466	56
Mar19	Chr8: 33,625,278–33,627,696	F: GAGCGATTCAGAGACCAGAGA R: CTTCCAGAGCACAACCATTACTT	566	56
Mar20	Chr8: 33,625,278–33,627,696	F: CTCTGGAAGCGTCTGAATGC R: CCAACCACAACTTAATCTGAACTAC	231	57
Mar21	Chr8: 33,626,278–33,626,696	F: AGCGCACTGCCTCTCATACT R: GCATTTCCCGACCTGTAAAA	358	57
Mar22	Chr8: 33,626,278–33,626,696	F: AGCGCACTGCCTCTCATACT R: CTTGTGAAATATGCCTCGCA	375	56
Mar23	Chr8: 33,626,281–33,626,686	F: AATGGTTGTGCTCTGGAAGCG R: CCTCGCATTTCCCGACCTGTAA	274	58
Mar24	Chr8: 33,626,781–33,627,375	F: CAAACACAAAAACGACACGC R: AAGCGCTTTGGGTGTACAGT	453	58
Mar25	Chr8: 33,626,781–33,627,375	F: TGTTTCATTCAGGCCTTCCT R: TGACTTTGACCTTTGACCCC	500	57
Mar26	Chr8: 33,626,781–33,627,375	F: GGATGGACTGATGATTCACTACAAC R: AAAGCGCTTTGGGTGTACAGT	412	57
Mar27	Chr8: 33,626,781–33,627,375	F: ACAGTTCCAGTTGTGTCTCCACTT R: TCTGATACGGCGCAATGAAGATCC	405	56
Mar28	Chr8: 33,626,816–33,627,324	F: AGTGATTCAGATTGGATGGACTGA R: AACACTATATCCTGCACCGGATT	509	56
Mar29	Chr12: 6,143,157–6,143,771	F: TGGAGATCTCAAGTGCTCTTCAAG R: ATGTGGTATTGCCTGGATGATCTC	451	58
Mar30	Chr14: 16,536,659–16,536,680	F: CCATAGCAACACCTTAGCAAACAC R: GGGCTTTGCAATGGTGGTTAAA	320	59
Mar31	Chr14: 16,536,836–1,653,710	F: CCAGTAACCACTCAGATCTTCTG R: TTAGTTGCTAGAGCATTGCTGTG	220	56
Mar32	Chr14: 16,563,469–16,563,648	F: TGGTTCATTCACACTGCCAGT R: TCCATGCGTGTTCACTATAGTCC	204	58
Mar33	Chr18: 17,827,232–17,827,798	F: TTGAACGACACTCTGCACCT R: TGAAGCTGAAAAAGCCACAA	837	58
Mar34	Chr18: 17,827,232–17,827,798	F: TTCTGCCAGTCTTTTCCTGG R: TTTGAACGACACTCTGCACC	497	57
Mar35	Chr18: 17,827,812–17,828,407	F: AAGCCATAGCCCCTTTTCAT R: ATCGGTGAGTCTGATACGGC	466	58
Mar36	Chr18: 17,827,812–17,828,407	F: ACAAAGCCATAGCCCCTTTT R: ATCGGTGAGTCTGATACGGC	469	59
Mar37	Chr20: 2,906,171–2,906,720	F: TCAATTTCTCATGCAGTTTGC R: AGCGTTGCACAAACACAAAG	402	59
Mar38	Chr20: 2,906,224–2,907,256	F: CTTTGTGTTTGTGCAACGCTGT R: ACACCATGTTGAAATGCTTCCAGT	302	59
Mar39	Chr20: 2,906,721–2,907,377	F: CATGCAGCATCTTCTCCAAA R: ACCAACAACCCAATAACCCA	387	57
Mar40	Chr20: 2,906,721–2,907,377	F: TCATTGAACCATCGGCTACA R: ACCAACAACCCAATAACCCA	462	58
Mar41	Chr21: 10,711,992–10,712,371	F: TGCACTTCTGTCAATAGATCCTCTT R: TCGGTCATCACCATCATCAGAT	401	59
Mar42	Chr38: 4,802,762–4,802,784	F: TCATCAGTCCACAGAACACTATCC R: ACCATAGGAAGCATCATTCGC	350	56
Mar43	Chr38: 4,867,346–4,867,835	F: ACATTCATTGGACAATCTTGTCGG R: AACAAGGAGTGGAATGAATTGCC	469	57
Mar44	Chr38: 4,955,599–4,955,622	F: TTATCTTGCTCCTTCATCCATCTCC R: ACTTAATGAAGACAGGAGGCAATGG	248	57
Mar45	Chr39: 5,164,306–5,164,730	F: AAGGAAACCGGTATGTGCTG R: TGTGCAAAAAGCAGTCTTGG	396	58
Mar46	Chr39: 5,179,355–5,179,609	F: TTTTGCAGCAAGAGCTTTCA R: TTGCCCTTGTGGTAATGACA	423	58
Mar47	Chr39: 5,179,385–5,180,010	F: GTGCCTTACAAGAATAATGCTCTGA R: CTTTGCCCTTGTGGTAATGACA	325	58
Mar48	Chr39: 5,179,610–5,180,093	F: TGTCATTACCACAAGGGCAA R: AGGTATGGGGAAGGTGGAGT	371	56
Mar49	Chr39: 5,179,610–5,180,093	F: TGTCATTACCACAAGGGCAA R: GGTGGAGTGCAGTGAGTCAG	359	56
Mar50	Chr39: 5,180,173–5,180,725	F: TGTCTTTGTTGCTGACCTTCA R: GGCCATCTCTTGTTGGAGTC	399	58

## Data Availability

The data used in the present study can be downloaded from the NCBI database under project number PRJNA1286929, https://www.ncbi.nlm.nih.gov/nuccore/?term=+PRJNA1286929 (accessed on 20 May 2025).
